# Factors that Affect Suicide Attempts of Adolescents in Multicultural Families in Korea

**DOI:** 10.3390/ijerph13121184

**Published:** 2016-11-28

**Authors:** Subin Park, Yeeun Lee

**Affiliations:** 1 Department of Research Planning, Mental Health Research Institute, National Center for Mental Health 127, Yongmasan-ro, Gwangin-gu, Seoul 04933, Korea; 2 Department of Psychology, Korea University, 145 Anam-ro, Seongbuk-gu, Seoul 02841, Korea; tasarang1010@gmail.com

**Keywords:** multicultural families, suicide, risk factor, adolescents

## Abstract

We examined the factors that affect suicide attempts adolescents multicultural families in South Korea. The participants were 727 adolescents whose mothers and/or fathers were born outside of South Korea (376 males and 351 females). Among them, 41 (weighted prevalence 6.2%) had attempted suicide during the last 12 months. Female gender, residence in large cities (compared with in rural areas), living with relatives/alone/with friends/in a dormitory or living in a facility (compared with living with family), high and low socio-economic status (compared with a middle level), high and low academic performance (compared with a middle level), severe perceived stress (compared with non-severe stress), conflicts with a teacher (compared with conflicts with parent), and foreign-father/-parent families (compared with foreign-mother family) were associated with increased odds of suicide attempt. The results indicate that greater awareness of the possibility of suicidal behavior is prudent for adolescents in multicultural families with certain risk factors, such as being from a foreign-parents family, living separately from the family, and having conflicts with a teacher.

## 1. Introduction

Suicide is a major global public health concern for children and adolescents [1]. World Health Organization (WHO) data indicate that suicide is the third leading cause of death among youth aged 15 to 19 years [2]. In 2013, there were 6.4 suicides per 100,000 population aged 15 to 19 years on average across the Organization for Economic Cooperation and Development (OECD) countries [3]. Suicide rates in the Republic of Korea (South Korea) are the highest of all OECD countries; among Korean adolescents in particular, these rates have constantly increased, in contrast to the decreased rates observed among adolescents of other OECD countries [3].

Since the mid-1990s, the numbers of multicultural families in South Korea has skyrocketed with the inflow of marriage-based immigrants and foreign workers [4,5,6,7]. According to the Ministry of Gender Equality and Family of the South Korea [6], in 2015 there were an estimated 278,036 multicultural families including marriage-based immigrants and naturalized citizens. In 2006, the number of middle and high-school students from such families was 1439; by 2015, this number had increased to 22,253 [8]. Non-professional workers and marriage-based immigrants make up the largest proportion of immigrants to South Korea and most of them are from East and Southeast Asian countries [9]. Although diverse policies and services are provided to these individuals via Multi-Cultural Family Support Centers, including education in the Korean language and culture, health care policies directed specifically at immigrants are relatively scarce [10].

Previous studies in South Korea have shown that multicultural families are more likely to have a lower socio-economic status, while children from multicultural families are more likely to experience school difficulties, be bullied, have low academic achievement, and display aggressive behavior and conduct problems [5,7,11]. During adolescence, difficulties in school, relational problems with peers, and social maladjustment might result in suicidal thoughts and self-destructive behavior [12]. Particularly, prior studies have found significant associations between increased suicide risk in adolescents and parental factors such as low-income and low family support [13], low academic performance [14], and school maladjustment (including interpersonal difficulties) [15,16]. Taken together, poor socio-economic status (e.g., low family economic status, residential type) and school maladjustment (e.g., low perceived academic achievement, interpersonal problems at school) might be associated with increased suicide risk among adolescents in multicultural families, compared to their peers with Korean parents.

In light of the high suicide rates in South Korea and rapid increase in the adolescent population from multicultural families, we investigated the factors that influence suicidal behaviors in a group of adolescents from multicultural families in South Korea.

## 2. Methods

### 2.1. Subjects

We used data from the 2015 Korean Youth Risk Behavior Web-Based Survey (KYRBS) [17]. The KYRBS has been performed annually since 2005 by the Korea Centers for Disease Control and Prevention, to monitor health behaviors among adolescents. The survey is conducted on a nationally representative sample of middle and high school students (12–18 years of age) and employs a stratified, clustered, multistage probability sampling design. Among the 70,362 students initially selected from 400 middle schools and 400 high schools, 68,043 (response rate 96.7%) ultimately participated in the survey. The KYRBS was reviewed and approved by the institutional review board of the Korea Centers for Disease Control and Prevention (2014-06EXP-02-P-A) [7,17,18].

Two questions were used to assess whether adolescents were from a multicultural family: “Was your father born in South Korea?” and “Was your mother born in South Korea?” Based on their answers, adolescents were classified into four groups: those whose father and mother were born in South Korea, those whose father was born in South Korea but whose mother was not (foreign-mother family), those whose mother was born in South Korea but whose father was not (foreign-father family), and those whose father and mother were both not born in South Korea (foreign-parents family) [7]. The latter three groups were classified as being from a multicultural family. Participants with absent fathers or mothers (i.e., children of single-parent families or orphans) were excluded from the study, because no further questions, such as birthplace, could be asked for the absent parent (i.e., dead or whose identity is not known). Among the 63,376 adolescents living with both parents (32,715 males and 30,661 females, mean age 15.06 ± 1.72 years), 727 (weighted prevalence 0.97%, 376 boys and 351 girls, mean age 14.85 ± 1.70 years) were from a multicultural family. The present study analyzed the data of these 727 participants. The birthplaces of the parents of participating adolescents are shown in Table 1.

### 2.2. Measurements

Suicidal behaviors were assessed using the question: “Have you attempted suicide during the past 12 months?” The socio-demographic variables assessed included sex, age, place of residence, residential type (living with family, with relatives/friends/alone/in a dormitory, or in a facility), and perceived family economic status and academic performance (both assessed by using a five-point Likert scale) [18]. Based on participants’ responses to these questions on perceived family economic status and academic performance, we categorized participants into one of three groups: low (response of 1); middle (2–4); or high (5). Perceived stress was measured with the following question: “Usually, to what degree are you stressed?” The response options were “not at all” (1), “not so much” (2), “a little bit” (3), “quite a bit” (4), and “very much” (5). Participants who chose “very much” (score 5) were classified as the “severe perceived stress” group. Participants were also asked to choose one major cause of stress. The response options included conflict with parents (e.g., interference, discrimination, excessive expectation, neglect, abuse), family circumstances (e.g., poverty, marital conflict, separation or divorce of parents), conflict with teachers, peer relational problems (e.g., violence, being bullied, conflicts with a friend, relationship with boyfriend/girlfriend), academic grades (e.g., examination, future career), health problems, and physical appearance.

### 2.3. Statistical Analyses

The characteristics of suicide attempters and non-attempters were compared using chi-squared tests for categorical variables and independent *t*-tests for continuous variables. Univariate logistic regression analyses were performed to examine the association between suicidal behavior and each of the risk and protective factors. Then, multiple logistic regression analyses were used to examine the association between suicidal behavior and each independent variable after controlling for potential confounders. The variables included in the multivariate model were selected based on their significance in the univariate analyses and the correlations between the variables. In all analyses, we took into account the sampling design parameters, weighting, clustering, and stratifying factors. The proportions of respondents′ characteristics were weighted according to each respondent′s probability of being selected for the sex-, grade-, and school-type-specific distributions in the region [19,20]. All analyses were performed using these weighted data. SPSS Statistics 21.0 (IBM Corp., Armonk, NY, USA) was used to perform all statistical analyses, and *p*-values of less than 0.05 were considered significant.

## 3. Results

Among the 727 subjects, 41 had attempted suicide in the past 12 months. The odds of reporting suicidal behavior among adolescents from multicultural families were about three times higher than were those of adolescents from Korean families (weighted prevalence 6.2% vs. 2.2%, odds ratio (OR) = 2.94, 95% CI = 2.80–3.01). There were significant differences in areas of residence, residential type, socio-economic status, academic achievement, perceived severe stress, major stressors, and type of multicultural family between suicide attempters and non-attempters (Table 2).

In the univariate regression analysis, residence in a large city (compared with residence in a rural area (OR = 9.92)), living with relatives/alone/with friends/in a dormitory or living in a facility (compared with living with family (OR = 4.96 and 22.91 respectively)), high and low socio-economic status (compared with a middle level (OR = 5.12 and 9.07), respectively), high and low academic performance (compared with a middle level (OR = 4.73 and 3.92), respectively), severe perceived stress (compared with non-severe perceived stress (OR = 4.47)), and being from foreign-father and foreign-parents families (compared with a foreign-mother family (OR = 4.97 and 12.73), respectively) were associated with increased odds of reporting suicidal behavior. With regard to the major stressors, compared with conflicts with parents, conflict with a teacher (OR = 14.62) was associated with increased odds of suicidal behavior, whereas stress related to academic grades was associated with reduced odds of suicidal behavior (OR = 0.17).

For the multivariate analysis, we selected the variables that were significant in the univariate analyses, and further investigated the correlations between the selected variables. Because residential type, area of residence, socio-economic status, and level of academic achievement were significantly correlated (*p* < 0.001), we included only residential type in the multivariate analysis and excluded the three other variables. Finally, four variables (i.e., sex, residential type, major stressor, and type of multicultural family) were included in the multivariate analysis, after taking multicollinearity into account. Here, female sex (adjusted OR (aOR) = 2.59), living in a facility (aOR = 6.26), conflict with a teacher (aOR = 11.35), and being from a foreign-parents family (aOR = 4.22) were independently associated with increased odds of suicidal behavior. The c-statistic for the multivariate model was 0.845 (Table 3).

## 4. Discussion

The present study examined the risk of suicidal behavior among adolescents from multicultural families and identified the risk factors. The 12-month prevalence of suicidal behavior among these adolescents was 6.2%, which was approximately three times the rate among adolescents from Korean families. The main risk factors of suicidal behavior were socio-demographic factors, perceived stress, and presence of several major stressors.

Female sex and living in urban areas was associated with increased odds of suicidal behavior, which has similarly been found among South Korean adolescents [21,22,23]. Furthermore, also consistent with the results from South Korean adolescents, we found that living without families [21] and low socio-economic status [23] were risk factors, suggesting that a lack of social resources might increase the risk of suicidal behaviour. Perceived academic performance was also related: students with high or low academic achievement tended to have a higher risk than did those with a middle level of achievement, which has also been found in a sample of South Korean adolescents [23]. This is perhaps because students who perceive that their academic performance deviates from the average might experience higher academic stress, and might be under pressure either to maintain their excellent results or to catch up with their peers.

The level of perceived stress was associated with increased odds of reporting suicidal behaviors, which is consistent with findings from other adolescent samples [13,21,24,25]. Furthermore, among participants who reported experiencing the various major stressors, those who chose conflict with teachers had significantly greater odds of suicidal behavior compared to those who chose parental conflict. Children and adolescents from multicultural families are more likely to be involved in school violence and delinquent and aggressive behaviors [7,26,27], which reflects their school maladjustment and conflicts with teachers. Considering the strong association of school-related factors with these adolescents′ overall psycho-social adaptation [28], special attention should be paid to school maladjustment problems.

Additionally, type of multicultural family was related to increased odds of suicidal behavior: adolescents with a foreign father or foreign parents had higher odds than did those with a foreign mother. This finding is compatible with previous findings that adolescents with foreign-born fathers or parents were more likely to experience social ostracism [29,30] and exhibit suicidal ideation [31]. This might be due to the differing ethnic backgrounds among foreign parents. In the present study, only 3.5% of the mothers in foreign-mother families were from non-Asian countries, whereas 38.8% of fathers in the foreign-father families were from non-Asian countries. Accordingly, adolescent children with foreign mothers are more likely to have a similar appearance to Koreans than are those with foreign fathers or parents, which might ease their assimilation. In addition, the adolescents from foreign-mother families are in a better position linguistically because they learn the Korean language within their family [7].

In the multivariate analysis, which included sex, residential type, major stressors, and type of multicultural family as predictors, we identified independent associations between each variable and suicidal behavior. Female sex was significantly associated with increased odds of suicidal behavior, even after controlling for the three other major risk factors. The association between living in a facility and suicidal behavior remained significant, which implies that the disadvantageous social environment of such adolescents from multicultural families might influence their suicidal behavior. Regarding multicultural family type, being from a foreign-parents family was independently linked with increased odds of suicidal behavior. Adolescents with foreign parents might experience greater difficulties in adjusting to society because of their greater dissimilarity in physical appearance and cultural norms compared with adolescents having at least one Korean parent. Conflicts with teachers were also significantly linked to increased risk of suicidal behavior, independent of the other variables.

This study had several limitations. First, the adolescents from multicultural families were classified according to their parents′ birthplaces because the KYRBS did not obtain information about parents’ ethnicity or nationality. Accordingly, the adolescents from foreign-parents family might have included Korean parents who were born in foreign countries [7]. Second, this study did not gather information about the birthplace of the adolescents. Third, suicidal behaviors, the levels of perceived stress, and major stressors were likely underreported because of socially desirable response tendencies in survey research. Fourth, this study lacked a comparison group such as Korean adolescents from non-immigrant families. Finally, although important implications might be derived from examining the interaction effects between the studied variables [32], the number of suicide attempters in our sample was too small for such an analysis.

## 5. Conclusions

Adolescents from multicultural families with specific risk factors, such as being from a foreign-parents family, living separately from the family, and having conflicts with a teacher, have a greater risk of suicide. As predicted, the lack of social resources and difficulty of school adaptation of children of multicultural parents might be major obstacles to their psychological adaptation in Korean society. Greater awareness of this possibility is needed. For these high-risk groups, preventive strategies might include stabilizing the living environment and reinforcing coping skills for stress and school maladjustment.

## Figures and Tables

**Table 1:** Birthplaces of parents in multicultural families.

Birthplaces	Foreign-Mother Family (*n* = 575), %	Foreign-Father Family (*n* = 67), %	Foreign-Parents family (*n* = 85), %	
	Mother′s Birthplace	Father′s Birthplace	Mother′s Birthplace	Father′s Birthplace
China (ethnic Koreans)	25.7	10.4	15.3	22.4
China (Han and other ethnicities)	15.5	6.0	11.8	11.8
Japan	27.7	34.3	4.7	2.4
North Korea	0.9	3.0	23.5	22.4
Other Asian countries	26.8	7.5	23.5	14.1
Non-Asian countries	3.5	38.8	21.2	27.1

**Table 2:** Characteristics of the study population (*n* = 727).

Variables	Total		Person with a History of a Suicide Attempt	Person without a History of a Suicide Attempt	*p*
	Before Adjustment n (%)	After Adjustment %	After Adjustment ^†^ %	After Adjustment ^‡^ %	
**Sex**					
**Male**	376 (51.72)	54.24	45.49	54.81	0.320
**Female**	351 (48.28)	45.76	54.51	45.19	
**Age, mean (SE)**	14.68 (1.71)	14.85 (0.08)	15.43 (0.39)	14.83 (0.08)	0.233
**Area of residence**					
**Rural**	91 (12.52)	9.78	1.49	10.32	0.004
**Small city**	371 (51.03)	48.99	40.85	49.53	
**Large city**	265 (36.45)	41.23	57.65	40.15	
**Residential type**					
**Living with family**	640 (88.03)	86.61	47.44	89.18	<0.001
**Living with relatives/ friends/alone/in a dormitory**	63 (8.67)	9.14	21.93	8.30	
**Living in a facility**	24 (3.3)	4.25	30.63	2.51	
**Socio-economic status**					
**High**	49 (6.74)	8.33	19.87	7.57	<0.001
**Middle**	616 (84.73)	81.96	43.31	84.50	
**Low**	62 (8.53)	9.71	36.82	7.93	
**Academic achievement**					
**High**	80 (11)	11.97	27.63	10.94	<0.001
**Middle**	533 (73.31)	70.97	38.97	73.08	
**Low**	114 (15.68)	17.06	33.40	15.98	
**Perceived severe stress**					
**No**	635 (87.35)	85.58	60.48	87.23	<0.001
**Yes**	92 (12.65)	14.42	39.52	12.77	
**Main cause of stress**					
**Conflict with parent**	99 (14.14)	14.55	13.79	14.59	<0.001
**Family circumstances**	47 (6.71)	6.45	15.59	5.94	
**Conflict with teachers**	23 (3.29)	3.04	25.01	1.81	
**Peer relational problems**	73 (10.43)	9.32	16.68	8.91	
**Academic grades**	343 (49)	49.88	8.53	52.18	
**Health problems**	34 (4.86)	5.30	7.60	5.17	
**Physical appearance**	81 (11.57)	11.47	12.80	11.40	
**Type of multicultural family**					
**Foreign mother**	575 (79.09)	74.23	27.25	77.32	<0.001
**Foreign father**	67 (9.22)	11.08	18.54	10.59	
**Foreign parents**	85 (11.69)	14.69	54.21	12.09	

^†^ Sample size = 41, weighted = 1875.15, ^‡^ Sample size = 686, weighted = 28,496.13.

**Table 3:** Odds ratios of predictors for suicidal behaviors in the past year among adolescents in multicultural families.

Variables	Univariate OR (95% CI)	Multivariate OR (95% CI) ^†^
Sex, female	1.45 (0.70–3.03)	**2.59 (1.06–6.36)**
Residential type		
Living with family	ref	ref
Living with relatives/friends/alone/in a dormitory	**4.96 (2.06–11.98)**	2.50 (0.93–6.73)
Living in a facility	**22.91 (8.43–62.28)**	**6.27 (1.82–21.62)**
Major stressor		
Conflict with parent	ref	
Family circumstances	2.78 (0.63–12.21)	1.69 (0.36–7.92)
Conflict with teachers	**14.62 (3.78–56.61)**	**11.35 (2.43–53.11)**
Peer relational problems	1.98 (0.50–7.88)	1.48 (0.33–6.59)
Academic grades	**0.17 (0.04–0.85)**	0.18 (0.03–1.02)
Health problems	1.56 (0.27–8.91)	2.00 (0.31–12.97)
Physical appearances	1.19 (0.27–5.27)	1.19 (0.21–6.84)
Type of multicultural family		
Foreign mother	ref	ref
Foreign father	**4.97 (1.64–15.04)**	1.94 (0.60–6.25)
Foreign parents	**12.73 (5.35–30.30)**	**4.22 (1.48–12.04)**
Age	1.24 (0.94–1.63)	
Area of residence		
Rural	ref	
Small city	5.70 (0.73–44.26)	
Large city	**9.92 (1.29–76.40)**	
Socio-economic status		
High	**5.12 (2.05–12.79)**	
Middle	ref	
Low	**9.07 (3.84–21.39)**	
Academic achievement		
High	**4.73 (1.95–11.48)**	
Middle	ref	
Low	**3.92 (1.66–9.28)**	
Perceived severe stress	**4.47 (2.21–9.03)**	

^†^ Multivariate odds ratios were calculated from multiple regression models including sex, residential type, main causes of stress, and type of multicultural family (c-statistic = 0.845), **bold** type, *p* < 0.05.
